# Effects of Warm-Mix Agents on the Thermal Stability of SBS-Modified Asphalt

**DOI:** 10.3390/ma19101970

**Published:** 2026-05-10

**Authors:** Qingdong Tao, Tianhong Xia, Desheng Yang, Hao Xiang, Ruizhe Si

**Affiliations:** 1 School of Architectural Engineering, Mianyang Polytechnic, Mianyang 621000, China; tqddll@126.com (Q.T.); xiatianhong0608@126.com (T.X.); 2 National & Local Joint Engineering Research Center of Transportation and Civil Engineering Materials, Chongqing Jiaotong University, Chongqing 400074, China; dshengyang@126.com; 3 Shock and Vibration of Engineering Materials and Structures Key Laboratory of Sichuan Province, Southwest University of Science and Technology, Mianyang 621010, China; 4 College of Civil Engineering, Southwest Jiaotong University, Chengdu 610031, China; ruizhesi@swjtu.edu.cn

**Keywords:** SBS-modified asphalt, warm-mix agent, thermal stability, dynamic shear rheology, microstructure

## Abstract

To evaluate the susceptibility of styrene–butadiene–styrene (SBS)-modified asphalt to modifier segregation during high-temperature storage, this study examined its segregation behavior and microstructural evolution under storage conditions ranging from 70 to 163 °C over durations of 48–144 h, with varying warm-mix agent dosages (0%, 3%, 4%, and 5%). The investigation was conducted using softening point measurements, dynamic shear rheometry, infrared spectroscopy, and optical microscopy. The results indicated that the incorporation of the warm-mix agent significantly reduced the difference in softening point, diminished the discrepancies in complex modulus and phase angle between the upper and lower layers, and inhibited SBS aggregation and phase separation. When the warm-mix agent content reached 5%, the softening point difference in the modified asphalt at 163 °C and 48 h decreased from 14.4 °C to 1.6 °C, essentially eliminating segregation. Infrared spectroscopy confirmed that the warm-mix agent did not induce chemical bond changes but improved the compatibility between the SBS modifier and the base asphalt. Microscopic observation further verified that the warm-mix agent facilitated a uniform dispersion of SBS modifier particles, forming a stable microphase structure. The research findings provide valuable insights for improving the storage stability and engineering performance of SBS-modified asphalt.

## 1. Introduction

In the field of road engineering construction, asphalt pavements have become widely applied due to their excellent driving comfort and convenient construction. As an indispensable key material, asphalt performance directly determines the service level of the pavement [1]. With increasing traffic loads and complex service environments, conventional base asphalt can hardly meet the demands of high-quality and long-life pavements. In this context, styrene–butadiene–styrene (SBS)-modified asphalt has become the most widely used binder in high-grade highways owing to its outstanding high-temperature stability, low-temperature cracking resistance, and durability [2]. However, SBS-modified asphalt faces critical challenges in production, high-temperature storage, and construction. The most prominent engineering issue is insufficient thermal storage stability. Long-term high-temperature storage easily causes separation of the SBS modifier from the asphalt matrix [3], weakening interfacial compatibility. This further leads to early pavement distresses including rutting, shoving, and cracking, seriously reducing service performance and life and limiting engineering application.

To solve the problem of insufficient thermal stability of SBS-modified asphalt, researchers have carried out extensive studies, mainly focusing on modifiers, stabilizers, and influencing factors. Islam et al. [4] emphasized that the performance of asphalt mixtures is closely related to modifier concentration. Li et al. [5] compared the effects of polyphosphoric acid, montmorillonite, and sulfur, and found that nano-montmorillonite provides the best comprehensive improvement on the storage stability of high-content SBS-modified asphalt. Li et al. [6] revealed the influence of base asphalt components, pointing out that a stable and compatible system with SBS is easier to form when the asphaltene content is less than 10% and the aromatic content is relatively high. Singh et al. [7] suggested that iso-wax, polyphosphoric acid (PPA), and high-vinyl SBS help maintain stable performance during storage and transportation. Liu et al. [8] and Zhang et al. [9] used softening point difference, complex modulus difference, and rheological parameter percentage difference as effective indicators for evaluating storage stability. From the viewpoint of storage mode, Xiao et al. [10] reported that tank truck storage improves the high-temperature performance and storage stability of SBS/crumb rubber composite-modified asphalt.

Although the above studies provide various methods to improve the thermal stability of SBS-modified asphalt, most existing studies focus on traditional stabilizers, modifier types, or storage processes, and rarely consider the ternary system of asphalt, SBS modifier, and warm-mix additive as well as energy consumption and environmental impact during construction. Warm-mix technology, with the advantages of energy conservation and emission reduction, has been widely concerned and applied. However, studies on the effects of warm-mix additives on the thermal stability, which is a key service behavior of SBS-modified asphalt, are still insufficient, especially the systematic understanding of the compatibility and micro-mechanism of the asphalt–SBS–warm-mix ternary system.

Given the above research gaps, this study takes SBS-modified asphalt as the research object and prepares the asphalt–SBS–warm-mix additive ternary system with different warm-mix dosages. Using segregation tests, dynamic shear rheology, infrared spectroscopy, and optical microscopy, the effects of warm-mix additives on the thermal stability of SBS-modified asphalt are analyzed, and the modification mechanism is revealed. The innovations of this study include clarifying the anti-segregation mechanism of the ternary system under high-temperature storage and determining the optimal warm-mix dosage. The results can provide a scientific basis for improving the engineering applicability of SBS-modified asphalt with high stability and energy-saving characteristics.

## 2. Raw Materials and Test Methods

### 2.1. Raw Materials

**Figure 1:**
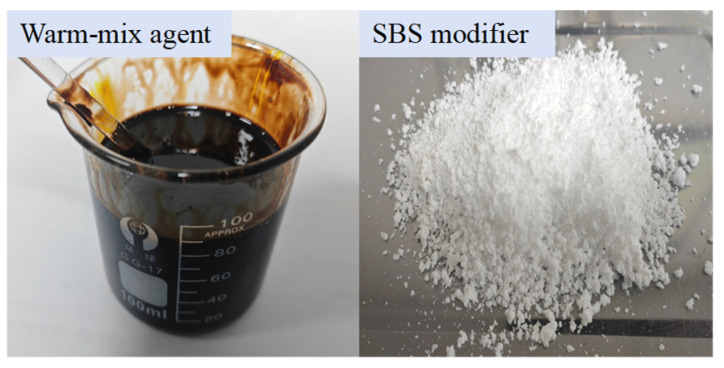
Raw Materials.

The SBS-modified asphalt used in this study was prepared on the basis of 70# asphalt. The SBS modifier with a block ratio of 30/70 was added at a content of 5%. The SBS-modified asphalt was first prepared by adding the SBS modifier for sufficient swelling, followed by the addition of the warm-mix agent. The warm-mix agent was composed of a methylstyrene block copolymer, appearing as a viscous brown liquid, as shown in Figure 1. It had a flash point of 190 °C, and its mass loss after heating at 135 °C for 72 h was less than 1.5%. The total content of benzene, toluene, xylene, and ethylbenzene was not greater than 0.1 mg/g. The warm-mix asphalt was prepared by shearing the warm-mix agent with the SBS-modified asphalt at 5000 r/min and 163 °C for 40 min using a high-speed shear mixer. The warm-mix agent dosages were 3%, 4%, and 5%, with the corresponding samples denoted by the suffix “#WA.” The technical specifications of each asphalt sample are shown in Table 1.

The specimen for the segregation test was prepared as follows: 50 g of asphalt was poured into a sample tube and then placed in an oven for quiescent storage. The storage temperatures were set to 70, 100, 130, and 160 °C, with storage durations of 48, 72, 96, and 144 h. After the storage period, the specimens were transferred to a refrigerator and cooled for 4 h. Subsequently, the upper and lower thirds of each specimen were cut and retained for testing.

### 2.2. Test Methods

#### 2.2.1. Softening Point Test

**Figure 2:**
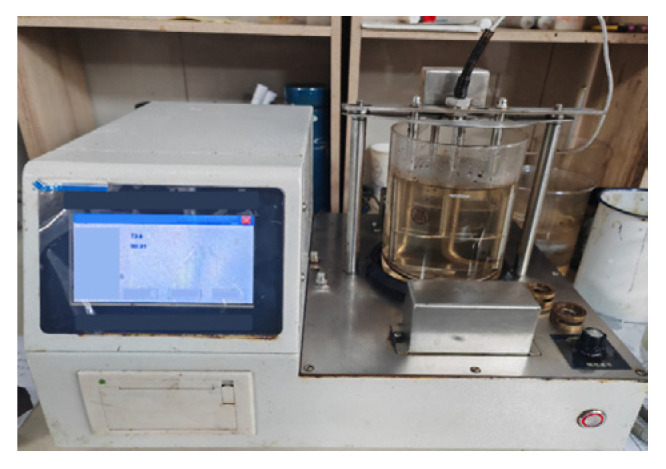
Softening point tester.

The softening point test was performed using a SYD-2806H automatic tester (Figure 2) using the ring-and-ball method (Shanghai Changji Geological Instrument Co., Ltd., Shanghai, China). The initial temperature of the heating medium was 5 °C. The temperature was recorded immediately when the softened sample dropped down to contact the surface of the lower plate, and the softening point difference was determined.

#### 2.2.2. DSR Tests

**Figure 3:**
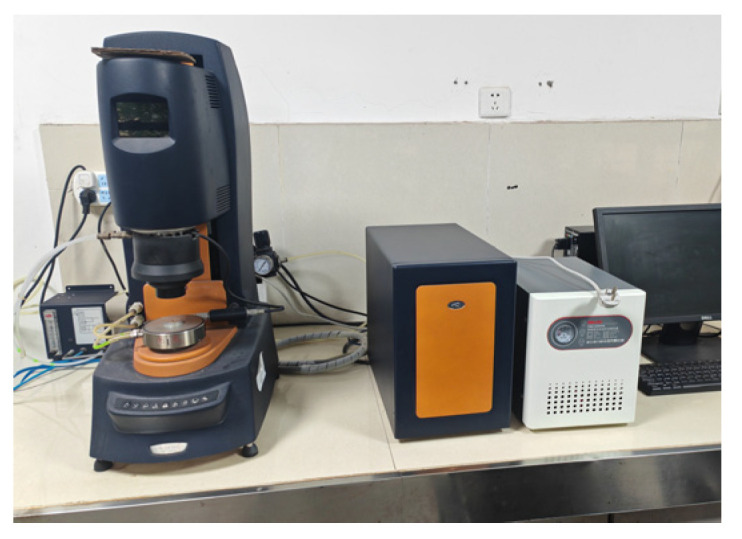
Dynamic shear rheometer.

Dynamic shear rheometry (DSR, Figure 3) tests were performed using a Discovery DHR-2 dynamic shear rheometer (TA Instruments, New Castle, DE, USA). The complex modulus and phase angle were obtained via a temperature sweep mode [11]. The specimen diameter was 8 mm and had a parallel plate gap of 2 mm. The test temperature ranged from 52 to 82 °C at a temperature gradient of 6 °C.

#### 2.2.3. FTIR Tests

**Figure 4:**
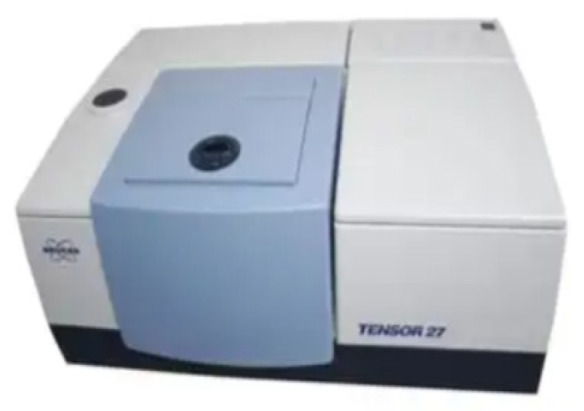
Infrared spectroscopy testing instrument.

Fourier transform infrared (FTIR, Figure 4) spectroscopy tests were conducted using a TENSOR-27 FTIR spectrometer (Bruker, Karlsruhe, Germany) with a scanning range of 500–4000 cm^−1^ and a resolution of 4 cm^−1^. Samples were prepared using the KBr pellet method to obtain infrared absorption spectra, and the distribution and difference in functional groups in different asphalt samples were analyzed [12].

To eliminate the interference of base asphalt in the evaluation of SBS modifier content, the evaluation was conducted according to the Equation (1).

(1) $$A=\frac{A_{966}}{A_{1377}}$$

where A is the standard index; a higher value of A indicates a higher content of the SBS modifier. $A_{966}$ and $A_{1377}$ are the characteristic peak areas at 966 and 1377 cm^−1^, respectively.

#### 2.2.4. Optical Microscopy Test

**Figure 5:**
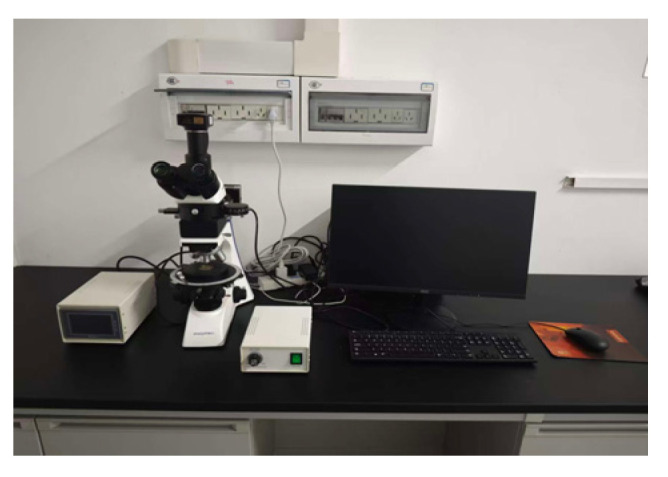
Optical microscope.

The mesostructure was observed using a BK-POL transmission polarizing microscope (Chongqing Optec Optical Instrument Co., Ltd., Chongqing, China), as shown in Figure 5. The microscope was equipped with an OTICS infinity color-corrected optical system and a 6 V/20 W halogen lamp transmission illumination system, enabling up to 600× magnification to observe the mesostructural evolution of asphalt at different temperatures [13].

## 3. Results and Discussion

### 3.1. Softening Point Test Results

**Figure 6:**
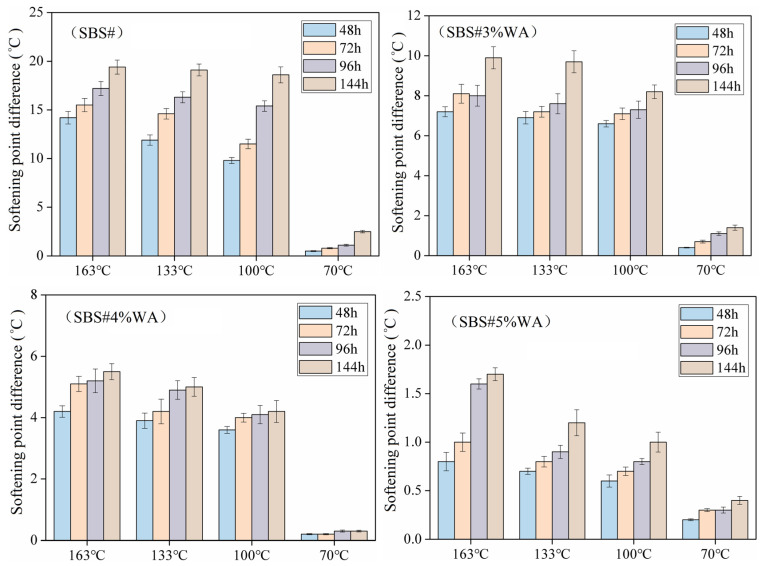
Softening point difference in asphalt after segregation.

Figure 6 shows the softening point differences between the top and bottom parts of asphalt specimens. After long-term high-temperature storage, the softening point differences between different layers of asphalt were apparent. Overall, the softening point difference in asphalt increased to varying degrees within the temperature range of 70–163 °C. The softening point difference in SBS# exceeded 10 °C under the time–temperature conditions of 48–144 h and 100–163 °C, which was significantly higher than the critical softening point difference for segregation (2.5 °C) given in the specification, indicating severe segregation of SBS# within this range.

Similar to the effect of temperature, prolonged storage time increased the softening point difference. At 133 °C, the softening point difference at 144 h increased by 60.5% compared with that at 48 h. This phenomenon was primarily attributed to the competition between the gravitational separation of the SBS modifier and base asphalt and the swelling of the SBS modifier by light components in asphalt at high temperatures. Gravitational separation causes segregation, whereas the swelling process restrains segregation [14,15].

Under gravity, the base asphalt, with a higher density, sank and the SBS modifier, with lower density, floated upward. However, the viscosity of asphalt provided resistance to the floating of the SBS modifier (e.g., SBS# at 133 °C), resulting in slight fluctuations in the softening point difference curve. When the temperature dropped to 100 °C, the SBS modifier agglomerated into flocs and particles, and the reduced swelling degree of asphalt to SBS modifier aggravated segregation [16].

The addition of warm-mix additives effectively reduced the softening point difference in asphalt. Taking 163 °C for 48 h as an example, the softening point differences were 14.2, 7.2, 4.2, and 0.8 °C at warm-mix additive contents of 0%, 3%, 4%, and 5%, respectively. This was because the aromatic components in the warm-mix additive enhanced the swelling degree of the SBS modifier. Meanwhile, the strong adsorption caused by high molecular potential energy between the SBS modifier and aromatic components filled the gaps in the SBS particle network, hindered the agglomeration of the SBS modifier, and made the modifier distribution more uniform.

### 3.2. DSR Test Results

#### 3.2.1. Rutting Factor

**Figure 7:**
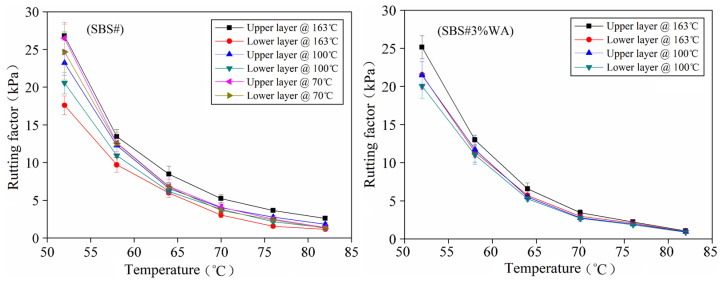

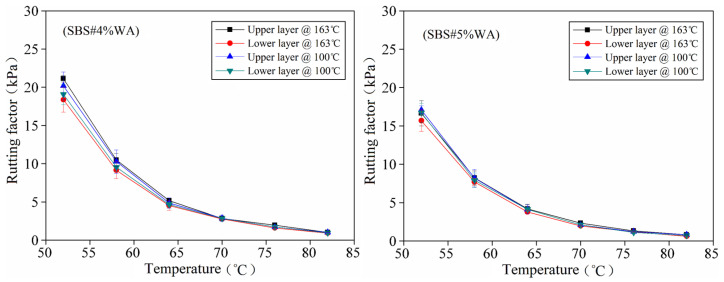
Rutting factor after asphalt segregation.

Figure 7 presents the test results of the rutting factor of asphalt. Generally, the rutting factor decreased with increasing temperature because the material exhibited apparent brittle and hard characteristics at low temperatures and a high deformation resistance.

The rutting factors of the upper and lower layers of the same asphalt were different, particularly when the test temperature was lower than 70 °C. After static storage under various conditions, the rutting factor of the upper layer of SBS# asphalt was higher than that of the lower layer. This was because the floating and agglomeration of SBS modifier led to a more cross-linked network structure in the upper layer, thus improving deformation resistance [17].

Note that no significant difference was observed in the rutting factor between the upper and lower layers of asphalt after static storage at 70 °C, indicating that the floating or precipitation of components in SBS# asphalt occurred remarkably above this temperature. At 70 °C and below, the cohesion and viscosity of SBS# asphalt restricted the thermal motion of components.

Taking a segregation temperature of 163 °C as an example, at the test temperature of 52 °C, the rutting factors of the upper layer were 26.8, 25.1, 21.1, and 16.7 kPa and those of the lower layer were 17.6, 21.5, 18.4, and 15.7 kPa, corresponding to warm-mix additive contents of 0%, 3%, 4%, and 5%, respectively. The differences between upper and lower layers were 9.2, 3.6, 2.7, and 1.0 kPa, respectively.

The incorporation of the warm-mix additive into SBS# asphalt reduced both the rutting factor value and the difference between the upper and lower layers, further verifying that warm-mix additive alleviated the segregation of SBS# asphalt.

#### 3.2.2. Phase Angle

**Figure 8:**
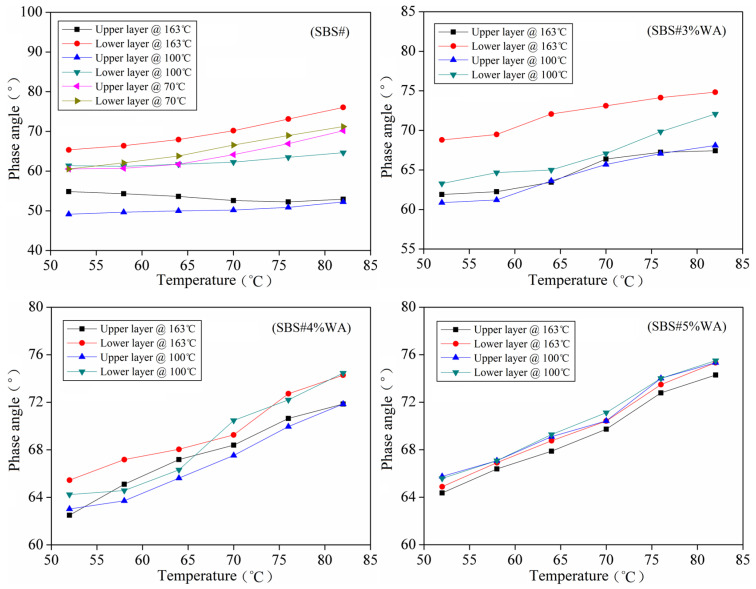
Phase angle after asphalt segregation.

The phase angle reflects the deformation resistance of asphalt at high temperatures. Figure 8 shows the phase angle test results of asphalt. Overall, the phase angle of asphalt is positively correlated with the test temperature.

An increase in temperature increased the viscous component and free volume of asphalt, intensified the motion of molecular chains, and promoted the transition of asphalt from a high-elastic state to a visco-fluid state. These changes enhanced the viscous behavior and weakened the elastic behavior of asphalt, resulting in an increased phase angle.

Significant differences in the phase angle were recorded between the upper and lower layers of asphalt. The phase angle of the lower layer was larger than that of the upper layer for the same asphalt, and the difference increased with the increase in static temperature. In this study, the average phase angle differences between upper and lower layers of SBS# asphalt after static storage at 70 and 163 °C were approximately 5° and 20°, respectively, indicating that elevated temperature accelerated phase separation and component migration in SBS# asphalt, enlarging the difference in polymer concentration between layers.

The SBS-rich layer was elasticity-dominated (low phase angle), whereas the SBS-poor layer was viscosity-dominated (high phase angle). Therefore, the phase angle difference became increasingly significant at higher temperatures.

When the warm-mix modifier was added, the phase angle difference between upper and lower layers decreased further. In particular, the phase angle difference in SBS#5%WA was less than 3°. This was because the dispersion effect of warm-mix additive enabled SBS molecular chains to distribute more uniformly in the asphalt, forming a stable microphase structure, which hindered the agglomeration and migration of SBS particles and thus mitigated phase separation. Consequently, the viscoelasticity of the upper and lower layers was generally consistent, and the phase angle difference decreased significantly [18,19].

### 3.3. Functional Group Test Results

**Figure 9:**
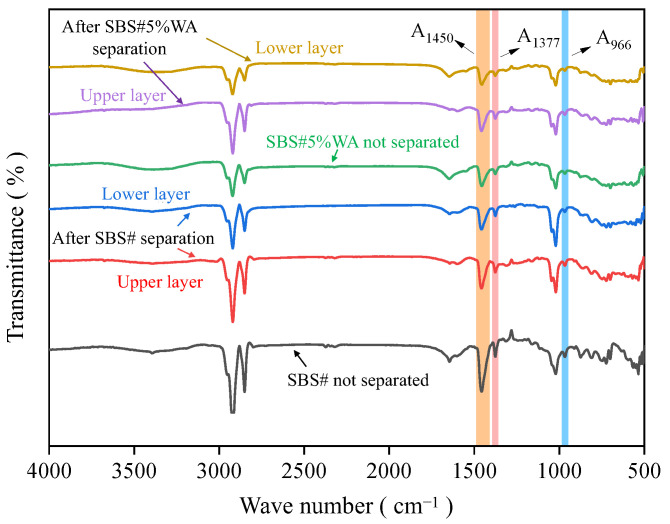
Infrared spectrum curve of asphalt.

To further investigate the thermal stability of the warm-mix-modified SBS# asphalt, FTIR was used to characterize the microstructure of the asphalt system. Figure 9 shows the FTIR spectra of the neat SBS#, the neat SBS#5%WA, and the upper and lower layers of SBS# and SBS#5%WA after storage at 163 °C for 96 h.

Figure 9 shows that within the wavenumber range of 500–4000 cm^−1^, the characteristic peaks of each asphalt appeared at 2920, 2850, 1450, 1377, 1020, and 966 cm^−1^. The positions of characteristic peaks remained unchanged after thermal segregation and the addition of warm-mix additive, indicating that no chemical reaction or new chemical bond formation occurred during the entire process.

The absorption peaks at 2920 and 2850 cm^−1^ were caused by the stretching vibration of -CH_2_-, and those at 1450 and 1377 cm^−1^ were attributed to the bending vibration of -CH_2_- and -CH_3_, respectively. These C-H stretching and bending vibrations of alkanes belonged to the characteristic peaks of base asphalt. The absorption peak at 966 cm^−1^ resulted from the C-H bending vibration of the trans-1,4-structure in polybutadiene (B segment) of the SBS modifier, which is the characteristic absorption peak of the SBS modifier [20,21].

**Figure 10:**
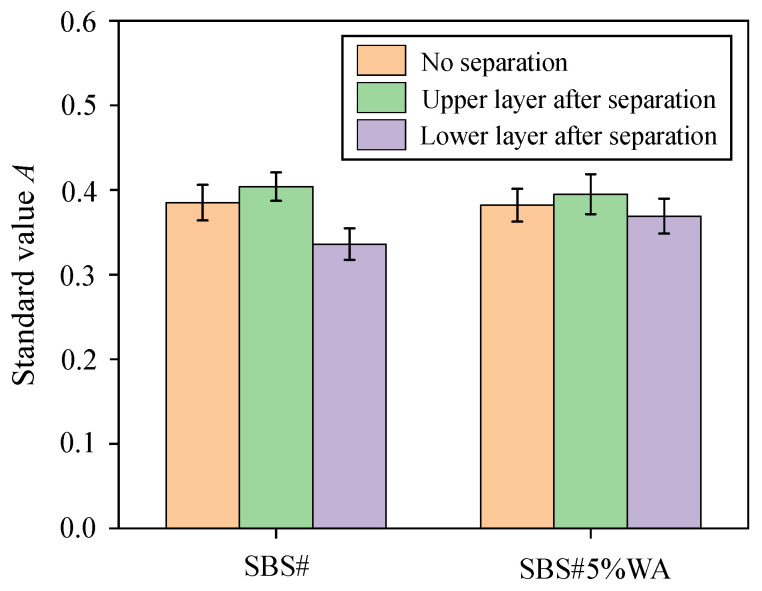
Standard value of SBS modifier content.

The ratio of characteristic peak areas at 966 and 1377 cm^−1^ calculated using Equation 1 is shown in Figure 10. The SBS modifier content of the two original asphalts (without segregation) was between those of the upper and lower layers after segregation. After segregation, the SBS modifier content in the upper layer was higher than that in the lower layer for both SBS# and SBS#5%WA, with standard value differences of 0.068 and 0.026, respectively. This further demonstrated that the addition of warm-mix additive contributed to restricting the segregation of SBS# asphalt.

### 3.4. Phase Structure Evolution Results

**Figure 11:**
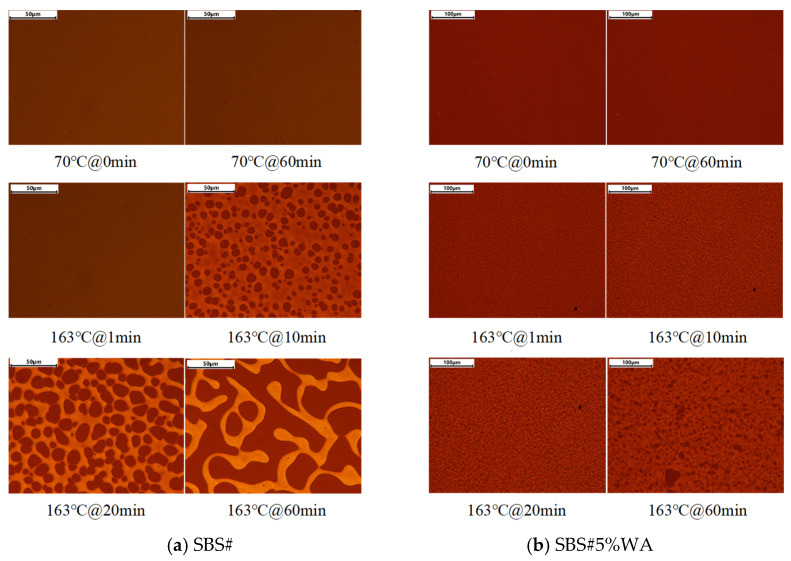
Microstructural evolution of asphalt segregation.

To further reveal the mechanism of warm-mix additive on the thermal stability of SBS# asphalt, we used optical microscopy with a heating stage to observe the microstructure evolution of asphalt. Figure 11 shows the test results of SBS# and SBS#5%WA at different temperatures over time.

As shown in Figure 11a, no apparent phase separation occurred in SBS# asphalt after 60 min at 70 °C, and the SBS modifier remained uniformly dispersed in asphalt to form a continuous phase. When the temperature increased to 163 °C, phase coarsening began in SBS# asphalt after 1 min. Significant phase separation appeared after 10 min, with SBS modifier particles of various sizes precipitating and dispersing in asphalt, indicating the occurrence of segregation. The maximum particle diameter of the SBS modifier was about 10 μm.

After 20 min, the asphalt exhibited a sea-island phase structure, and the SBS modifier particles fused, leading to increased particle size, whereas the particles were still uniformly dispersed in the continuous asphalt phase. After 60 min, the SBS particles melted completely to form a flowing phase with certain fluidity. Because the fluidity of asphalt was higher than that of the SBS modifier, the continuous asphalt phase surrounded the SBS modifier to form an irregular sea-island structure, at which point segregation was most severe.

As shown in Figure 11b, no apparent phase separation was observed in SBS#5%WA at either 70 or 163 °C. The SBS modifier was uniformly dispersed in the continuous asphalt phase. Only a slight phase coarsening occurred after 10 min at 163 °C. After 60 min, only a very small amount of SBS modifier precipitated and dispersed uniformly in the continuous asphalt phase. This indicated that the addition of the warm-mix additive improved the compatibility between the SBS modifier and base asphalt, thus reducing the segregation degree of SBS# asphalt at high temperatures [22].

The warm-mix agent can enhance the swelling degree of the SBS modifier, improve the interfacial compatibility between SBS and base asphalt, and inhibit the agglomeration and migration of SBS particles, thus improving the thermal stability of SBS-modified asphalt.

## 4. Conclusions

(1) Temperature and storage time have significant effects on the segregation behavior of SBS-modified asphalt. Severe segregation easily occurs under high-temperature and long-term storage conditions and is characterized by a remarkable increase in the softening point difference between upper and lower layers. The influence of temperature is more significant than that of time. Below 70 °C, the cohesion of asphalt is strong and the segregation degree is low.
(2) A warm-mix additive can effectively improve the thermal stability of SBS asphalt. When the warm-mix additive content is increased, the softening point difference, rutting factor difference, and phase angle difference between upper and lower layers all decrease significantly. The segregation degree is the smallest at a 5% warm-mix additive content, primarily because the aromatic components in warm-mix additive enhance SBS swelling and inhibit agglomeration, thus improving the uniformity and stability of the system. When the dosage exceeds 5%, excessive warm-mix agent may reduce asphalt viscosity and high-temperature performance.
(3) No new characteristic peaks or chemical bonds are formed when a warm-mix additive is added or thermal segregation is experienced. A warm-mix additive enhances the swelling degree of the SBS modifier, promotes the uniform dispersion of SBS molecular chains to form a stable microphase structure, hinders the agglomeration and migration of SBS particles, and strengthens the compatibility between the SBS modifier and base asphalt. Thus, the high-temperature segregation of asphalt is reduced.

## Figures and Tables

**Table 1:** Technical indexes of asphalt.

Asphalt Type	25 °C Penetration /0.1 mm	Softening Point /°C	Ductility @ 5 °C/cm	Brinell Viscosity @ 135 °C/(Pa·s)
SBS#	56.6 ± 1.2	87.0 ± 0.6	34.1 ± 2.1	2.445 ± 0.082
SBS#3%WA	62.6 ± 1.3	86.8 ± 0.5	44.0 ± 2.4	2.091 ± 0.077
SBS#4%WA	74.1 ± 1.5	84.5 ± 0.7	50.2 ± 2.6	1.783 ± 0.069
SBS#5%WA	80.4 ± 1.6	83.7 ± 0.5	62.8 ± 3.2	1.588 ± 0.063

## Data Availability

The original contributions presented in this study are included in the article. Further inquiries can be directed to the corresponding author.
